# A Step in Advance

**Published:** 1889-09

**Authors:** 


### A STEP IN ADVANCE.

THE sixth annual session of the National Association of Dental Faculties was held at Saratoga Springs, N. Y., August 5, 6, and 7, 1889.

This proved to be one of the most important sessions of the association since its organization. Although at first organized as a delegate body, the requirements for representation had became lax until last year, when it was found that few colleges had sent duly authorized delegates to represent them in the deliberations of the association. Steps were taken at that meeting to insure against such a contingency the present year, and with the effect that out of the twenty-six colleges, members of the National Association of Dental Faculties, the following twenty were represented by authorized delegates:

Baltimore College of Dental Surgery, R. B. Winder.
Boston Dental College, J. A. Follett.
Chicago College of Dental Surgery, Truman W. Brophy.
Columbian University, Dental Department, J. Hall Lewis.
Dental Department of Southern Medical College, L. D Carpenter.
Dental College of University of Michigan, J. Taft.
Harvard University, Dental Department, Thos. Fillebrown.
Indiana Dental College, J. E. Cravens.
Kansas City Dental College, J. D. Patterson.
Louisville College of Dentistry, J. Lewis Howe.
Missouri Dental College, W. H. Eames.
New York College of Dentistry, Frank Abbott.
Ohio College of Dental Surgery, H. A. Smith.
Pennsylvania College of Dental Surgery, C. N. Peirce.
State University of Iowa, Dental Department, A. O. Hunt.
University Dental College, J. S. Marshall.
University of Maryland, F. J. S. Gorgas.
University of Pennsylvania, Dental Department, James Truman.
Vanderbilt University, Dental Department, W. H. Morgan.

The Philadelphia Dental College, the Dental Department of the National University, the School of Dentistry of Meharry, Medical Department of Central Tennessee College, and the University of California, Dental Department, were not represented.

All the plans had been well laid for decisive work and all seemed to feel that an important step was to be taken. The attendance of seventeen colleges was necessary to constitute a quorum, and if at any time there had been a desire upon the part of any institution to block the wheels of legislation, it could easily have been done by withdrawing, as a bare quorum was present at any one sitting; but to the honor of the men who constituted the body, they were willing to abide by the honest decision of the association, and no filibustering was indulged in. Three vital questions were to be considered, and the discussion at times waxed warm.

At Monday's session credentials were examined and an organization effected. To facilitate business all the resolutions and amendments offered last year, which, under the constitution, had to wait a year, were now laid on the table.

On Tuesday, Dr. Truman moved to take from the table Dr. Howe's resolution of last year and consider the first clause,—viz., "That attendance upon three full regular courses, in separate years, be required before examination for graduation." A warm discussion followed, in which the representatives of the Southern dental colleges took a firm stand against the movement, basing their argument upon the ground that it was premature; that the medical colleges in their part of the country, at least, required only two years of five months each to make a physician, and that until such time as their course was lengthened any extension of the dental course would militate against the dental colleges and drive students into medicine instead of dentistry. The other side of the question was upheld as warmly by its adherents; but the hour growing late, with no probability of a vote's being reached that evening, a motion to adjourn was entertained, and the members retired to sleep over the question.

When the question was brought up the next morning and the roll called, it was found that eighteen delegates responded. The discussion was renewed with considerable ardor, the following gentlemen participating: Drs. Marshall, Abbott, Follett, Carpenter, Allport, Truman, Peirce, Morgan, Patterson, Sudduth, Gorgas, Taft, Brophy, Chandler. A vote finally having been reached, the count showed twelve for extension of time and six against. The six negative votes were afterwards cut down to four by a change in the vote of the Dental Department of the University of Maryland and the Southern Medical College, leaving the vote fourteen to four. On the next clause of the resolution, relating to length of term, the vote stood eighteen to one in favor of five months, the one vote was for a longer term.

The next question taken up was as to when the new rule should go into effect, and this was set for the beginning of the session of 1891 and 1892.

A motion was then entertained and passed, requiring the colleges belonging to the association to publish in their announcements for 1890–91 the action of the association regarding the extension of the course. This was done to avoid all confusion in regard to the matter.

The utmost good feeling predominated, and all seemed to fall into line without any reservation, which was one of the most assuring features of the movement. Twenty out of the twenty-five active colleges recognized by the National Association of Dental Examiners, are now bound by their vote to adopt the new rule, and the other four, which have made application for membership, had pledged themselves in advance of the action of the association to support a three-years' course. One feature to be noted is that nearly all the younger institutions were ardent advocates of the need of lengthening the course.

The National Association of Dental Faculties has done a most excellent work in the five years of its existence, and stands a fitting rebuke to those medical colleges of our country which are still holding back in the onward movement of scientific education. The dental profession is to-day better organized than any other of the learned professions in this or in any country, with its colleges united into one harmonious association, working for the upbuilding of the standard of the specialty which they teach. With statutory enactments in a large majority of the States, and a thoroughly organized national association of dental examiners, consisting of delegate members from the boards of examiners of the several States that have laws governing the practice of dentistry, we may well be proud of our position, and respectfully challenge the medical profession to emulate our example. The dental colleges could not have taken a step better calculated to establish it undeniably among the learned professions than the one just adopted. With a three-years' graded course of instruction and with preliminary and intermediate examinations, they present to the world as good standing as can be claimed by any institution in this country. Whether dentistry is ever recognized as a specialty of medicine or not, she is fast making strides towards perfection in her own ranks that will entitle the degree of D.D.S. to as much consideration as any degree granted in this country, and when we come to consider her youth and her wonderful achievements, we may well exclaim, "What a precocious child!"

The action of the association is sure to receive favorable remark from the medical press. The first we have observed comes from the *Times and Register*, Philadelphia, which we take pleasure in copying. After stating what action had been taken the editorial says,—

"It will thus be seen that the dental profession has taken a decided step in advance, one which places it ahead of the profession of medicine, and in fact puts the latter into the position of requiring less time for the study of the whole science and art of healing than modern dentists demand for the acquiring of a small portion of the same art. The action of the association will be endorsed by all the dental colleges. In fact, they will be practically compelled to do so, as dental boards exist in many States, which are united in an Association of Dental Examiners. This body has the power of forcing any recalcitrant college into conformity with its requirements by refusing to recognize its diplomas without examination. A Baltimore college defied the association for one year, but was glad to come into the fold before the opening of the next term.

"The Association of Dental Faculties has been in existence for five years, and in that short period has accomplished three decided reforms in dental education. First, it put a stop to the practice of allowing practitioners of five years' standing to enter the graduating class. Then it established the graded course, with preliminary and junior examinations; and, finally, it has instituted the great reform herein recorded. One result of this action will be to increase by one-half the cost of the dentist's course of instruction, as the same body regulates the fees to be charged at dental colleges, and the student has now to pay for three terms.

"It will thus be seen that the dental profession is much better organized than the medical, and that this is made possible largely through the influence of legislative enactments; further, that this has made possible an advance in dental education, which could not have been secured otherwise, as the weaker schools would have held back those desirous of lengthening the course. The difficulties in the way of procuring suitable legislation are much less in dentistry, as there are no homœopathic or eclectic dentists."

The following officers were elected for the ensuing year:

James Truman, President; L. D. Carpenter, Vice-President; J. E. Cravens, Secretary; A. W. Harlan, Treasurer; Frank Abbott, J. Taft, and F. J. S. Gorgas, Executive Committee.

The following committees were appointed: *Ad Interim* Committee, Drs. T. W. Brophy, R. B. Winder, and J. A. Follett; Committee on Schools, Drs. H. A. Smith, J. D. Patterson, J. Lewis Howe, W. H. Morgan, W. H. Eames.

Adjourned to meet at the call of the Executive Committee.

